# Surgery in Bilateral Wilms Tumor—A Single-Center Experience

**DOI:** 10.3390/children10111790

**Published:** 2023-11-07

**Authors:** Fernanda Kelly Marques de Souza, Mayara Caroline Amorim Fanelli, Alexandre Alberto Barros Duarte, Maria Teresa de Seixas Alves, Henrique Manoel Lederman, Monica dos Santos Cypriano, Simone de Campos Vieira Abib

**Affiliations:** 1 Department of Pediatric Surgery, Pediatric Oncology Institute, GRAACC, Federal University of São Paulo, São Paulo 04039-001, Brazil; mayara.amorim76@gmail.com (M.C.A.F.); aabd@uol.com.br (A.A.B.D.); simoneabib@uol.com.br (S.d.C.V.A.); 2 Department of Pediatric Surgery, Foundation Regional Faculty of Medicine of São José do Rio Preto, Children’s and Maternity Hospital, São José do Rio Preto 15091-240, Brazil; 3 Department of Pathology, Federal University of São Paulo, São Paulo 04023-062, Brazil; mtsalves@unifesp.br; 4 Department of Radiology, Pediatric Oncology Institute, GRAACC, Federal University of São Paulo, São Paulo 04039-001, Brazil; henrique.lederman@graacc.org.br; 5 Department of Pediatric Oncology, Pediatric Oncology Institute, GRAACC, Federal University of São Paulo, São Paulo 04039-001, Brazil; monicacypriano@graacc.org.br

**Keywords:** bilateral Wilms tumor, nephron-sparing surgery, renal tumor, renal surgery

## Abstract

The treatment of bilateral Wilms tumors (BWT) involves curing the cancer, preserving long-term renal function, and maintaining a good quality of life. Established methods for achieving these goals include preoperative chemotherapy and nephron-sparing surgery (NSS). This study aimed to evaluate the experience of a single institution in treating patients with BWT. We analyzed cases of BWT treated at the Pediatric Oncology Institute—GRAACC—Federal University of São Paulo over a period of 35 years. Bleeding control was performed with manual compression of the renal parenchyma. Thirty-three patients were included in the study. Thirty cases were synchronous tumors. The mean age at diagnosis was 30.4 months (±22 m) and 66.7% were girls. The median follow-up period was 83 months. Neoadjuvant chemotherapy was the primary approach in most patients (87.9%), with a simultaneous upfront surgical approach performed in 84.8%. Most patients underwent bilateral NSS (70.4%). There were no early complications in this series, but 39.4% had clinical complications. The five-year survival rate was 76%. Therefore, it is clear that the surgical approach to BWT plays a crucial role in achieving good outcomes. However, it is difficult to standardize surgical techniques and technology may have the potential to enhance safety.

## 1. Introduction

Wilms tumor is the most common primary malignant tumor of the kidneys in childhood, accounting for more than 95% of kidney tumors [1,2]. However, bilateral cases are rare, with an incidence of 5% of synchronous tumors [3], and 2–3% of metachronous cases [4].

Bilateral cases have an earlier age of onset compared to unilateral cases and are associated with lower 5-year survival rates of 61–80.8% versus over 85% for unilateral cases [5,6]. Factors such as the use of nephrotoxic drugs, radiation, and associated syndromes that predispose to renal failure (Beckwith–Wiedemann Syndrome, Denys–Drash Syndrome, Sotos Syndrome, Perlman Syndrome, WAGR, hemihypertrophy, aniridia) [7], and genetic predisposition to metachronous disease may also influence prognosis and must be considered in surgical planning [8].

Surgery is a critical component of treatment for bilateral Wilms tumors, which is challenging, not only regarding the surgical strategy but also due to treatment goals of preserving renal function and avoiding complications related to dialysis and kidney transplantation [9]. To achieve these goals, preoperative chemotherapy and nephron-sparing surgery are well established both in COG (Children’s Oncology Group) and SIOP (International Society of Paediatric Oncology) protocols [10].

Neoadjuvant chemotherapy can reduce tumor volume and form a pseudo-capsule around tumors, allowing for less extensive surgery without compromising outcomes [11,12]. From a surgical point of view, renal vessels are fragile and prone to spasms, so a surgical technique that avoids manipulating them can be considered safer.

Various surgical techniques and technology, such as intraoperative ultrasound, minimally invasive approaches, 3D modeling, fluorescence-guided surgery, spectroscopy-guided surgery, radio-guided surgery, photodynamic therapy, and near-infrared photoimmunotherapy, can help the surgical team decide on preservation and facilitate surgical procedures [13,14].

We aimed to evaluate a single institution’s experience in treating patients with bilateral Wilms tumors and critically analyze the surgical aspects of their treatment.

## 2. Materials and Methods

We conducted a retrospective analysis of patients’ records of bilateral Wilms tumors treated at the Pediatric Oncology Institute—GRAACC—Federal University of São Paulo from December 1987 to September 2022. Descriptive statistical analysis was performed.

On average, the institution receives twelve cases of Wilms tumors per year, and 18% of these are bilateral.

### Surgical Planning and Technique

We use a single approach to operate on both kidneys. After complete mobilization and analysis of both kidneys, the kidney with the largest tumor volume is always approached first. Nodulectomies, partial nephrectomies, or nephrectomies are indicated depending on the tumor locations and relation to the renal hilum. Cautery delimitation of the parts to be resected is made and the tumors are resected. Since surgical margins do not impact prognosis [15,16], it is recommended to be conservative in resection, so that a greater percentage of renal parenchyma can be preserved. Despite this fact, margins were identified in pathological analysis. Bleeding control is made with manual compression of the renal parenchyma, reducing injury to the remaining parenchyma and to the vascular elements of the renal hilum. We do not clamp the elements of the hilum, nor do we use vascular loops to isolate them, to avoid injury or vascular spasm. Hemostasis of greater parenchymal vessels are made with sutures and cautery. The manual compression was efficient. The perioperative estimated blood loss was not significant and did not determine hemodynamic instability.

**Figure 1:**
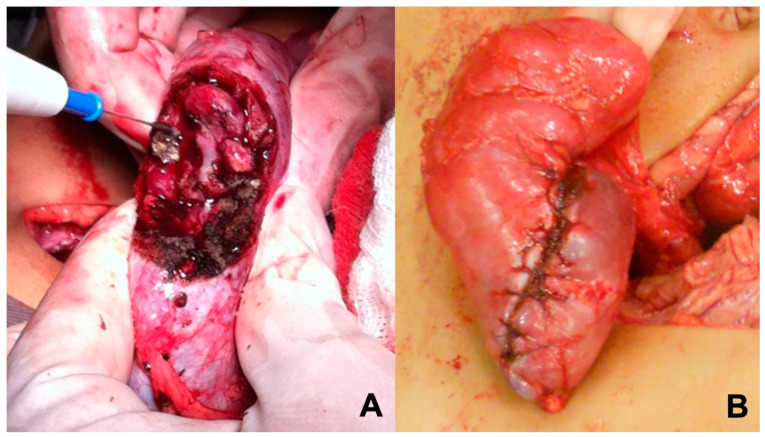
Surgical technique. (**A**) Manual compression of the renal parenchyma for bleeding control; (**B**) Reconstruction with “wrapping the parenchyma”.

Reconstruction of the remaining parenchyma includes closure of renal calyces and “wrapping the parenchyma” (Figure 1) both for hemostasis and urinary fistula prevention. Cooling of the surgical field, preservation solutions, ultrasonic power devices, hemostatic agents, or bench surgery were not used in this series. Each kidney should be staged separately, and it is important to sample lymph nodes on both sides and report accordingly. Penrose drainage and ureteral stenting were avoided in most cases. The indication for intraoperative dialysis catheter implantation was reserved only for cases when the patient became anephric.

## 3. Results

**Figure 2:**
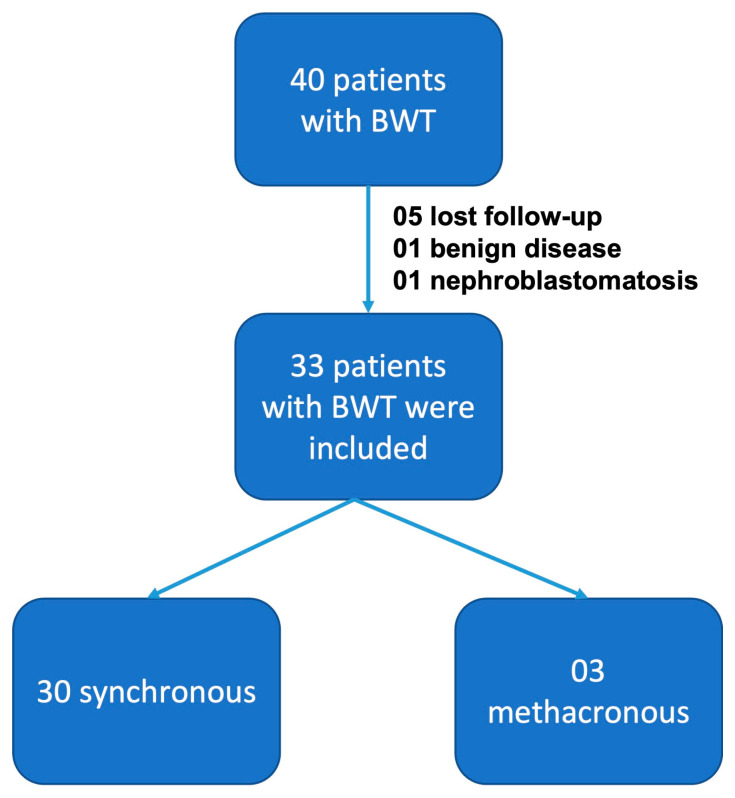
Patients included in this study.

Forty children were diagnosed and treated for bilateral Wilms tumor at the Pediatric Oncology Institute—GRAACC—Federal University of São Paulo. Among these, seven patients were excluded: five due to loss of follow-up, one due to a diagnosis of benign disease, and one due to a diagnosis of bilateral nephroblastomatosis with no finding of nephroblastoma on biopsy. Thirty cases were synchronous tumors, and three patients had metachronous tumors (Figure 2). The mean age at diagnosis was 30.4 months (±22 m) and 66.7% were girls.

Patients’ characteristics are outlined in Table 1. At diagnosis, four patients (12.1%) had metastases, one in the liver and the other three in the lungs. However, all of them regressed with neoadjuvant chemotherapy. Vascular extension was diagnosed in one case (3%) in the inferior vena cava, which completely shrunk with preoperative chemotherapy. The median follow-up was 83.3 months. Neoadjuvant chemotherapy was the first approach in most patients (29 patients, 87.9%). Simultaneous surgical approach was performed in 84.8% and most patients underwent bilateral nephron-sparing surgery (70.4%). Staged procedures were made only for patients who were operated on elsewhere as the first approach, two of whom were submitted to total nephrectomy in the most affected side before coming to our hospital.

**Figure 3:**
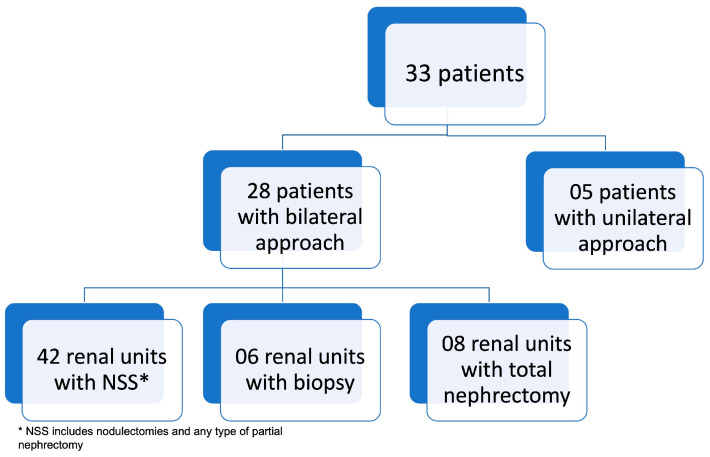
Approach to renal units in the first procedure.

Eight patients (24.2%) underwent biopsy before surgery, five before 2001, when the treatment was carried out according to the NWTS (National Wilms Tumor Study) and GBTW (Wilms Tumor Brazilian Group) protocols, and three patients after neoadjuvant chemotherapy due to no response to chemotherapy, according to the former SIOP (International Society of Paediatric Oncology) protocol. Patients underwent different surgical procedures depending on the location and size of the tumor. The number of procedures per patient ranged from 1 to 5. Most patients underwent only one surgical procedure (57.6%). Radiotherapy was indicated in 54.5% of patients. A total of 61 renal units were addressed in the first surgery (Figure 3). Forty-three (70.5%) were treated with nephron-sparing surgery, thirty-one being classified according to the surgical technique as NSS (B). Fourteen (22.9%) received total nephrectomies and four (6.5%) only biopsy. A transverse incision was performed in 90.9%.

A Penrose drain was used in only three cases, one of which had a resection of 2/3 of the kidney, and reimplantation of the ureter in the upper calyx. There were no urinary fistulas in this series, nor any other early surgical complications. Thirteen patients (39.4%) had clinical complications, including two patients with chronic renal failure (CRF) due to bilateral nephrectomy, who died due to disease progression. Three patients developed acute renal failure after surgery, one of which was completely reversed with clinical measures, and another evolved with improvement in renal function after temporary dialysis. The third had CRF due to bilateral nephrectomy. Dialysis catheters were not routinely necessary in the immediate postoperative period, except for patients who underwent bilateral nephrectomies. Finally, eleven patients had systemic arterial hypertension in the postoperative period, with one of them requiring long-term clinical treatment.

Staging data were retrieved in 46 kidneys only and histology in 51: 09 stage I, 25 stage II, and 12 stage III. Histological risk was high in 9 and intermediate in 42 kidneys. Information about lymph nodes was lacking. Blastomatous histopathology was found in four of these patients. 

**Figure 4:**
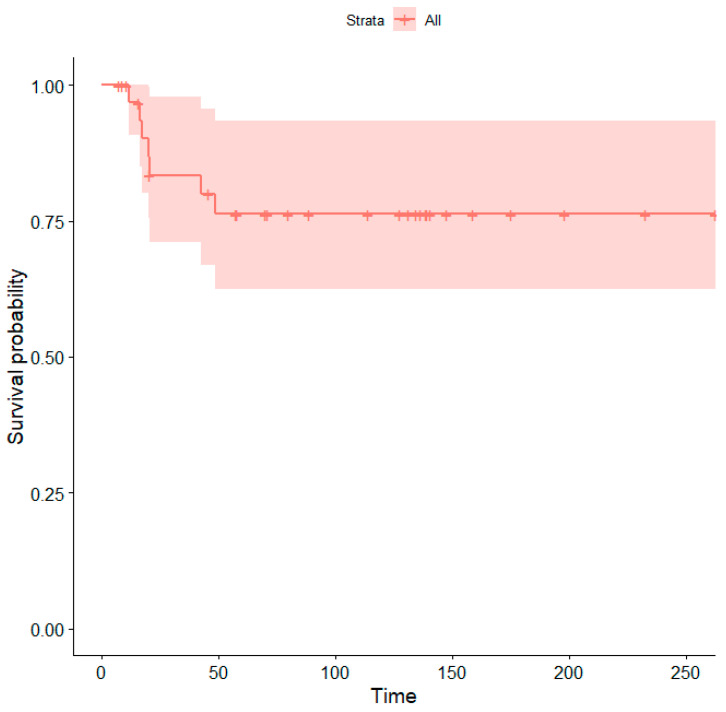
Kaplan–Meier curve showing overall survival probabilities (Time = time in months). The shaded area represents the 95% confidence interval.

The median follow-up was 83 months. Seven patients (21.2%) died due to disease progression. The five-year survival rate was 76% (Figure 4).

## 4. Discussion

Bilateral Wilms tumor presents unique epidemiological and treatment-related characteristics that have been thoroughly investigated over the years, resulting in improvements in survival rates and quality of life [17,18].

Previous studies report bilateral tumors at an earlier age. In this study, we found that the median age at diagnosis was 20.5 months, which is slightly earlier than the range of 24–32 months reported in the literature [3,19,20,21]. Additionally, most cases (59%) occurred in girls, consistent with previous findings [10,22]. Abdominal mass is the most frequent sign of both unilateral and bilateral Wilms tumors [23], and it was the most prevalent initial symptom in our series, often accompanied by an increase in abdominal volume. Furthermore, the incidence of patients with associated malformations found in this study was similar to that described in the literature, ranging from 8 to 17% [24].

All current treatment protocols for bilateral Wilms tumors include preoperative chemotherapy to reduce tumor volume and allow a more conservative surgical approach, capable of preserving as much healthy renal tissue as possible. This approach aims to reduce the morbidity and mortality of these patients [10,25,26]. Before the advent of multicenter study groups, all patients with bilateral Wilms tumors underwent bilateral nephrectomies, with subsequent CRF, as it was believed that the only treatment option was initial aggressive surgical resection, which resulted in low survival [27].

In Brazil, anephric patients are only eligible for kidney transplantation after being two years out of treatment. None of our patients in that situation reached two-year survival.

However, the rate of renal failure has shown a significant decrease over the years. In the NWTS 1 and 2 studies, the rate of CRF was 16.4% for unilateral tumors, evolving to 9.9% in NWTS 3 and 3.9% in NWTS 4. Currently, the rate remains at around 0.6% for unilateral tumors and 11.5% for bilateral tumors, with 9% for synchronous tumors and 18% for metachronous ones. Warady et al. suggest that 10% of bilateral tumors are associated with chronic renal failure, compared to 0.7% of unilateral cases [28]. Total nephrectomy on the most affected side and conservative surgery on the contralateral kidney are present in a higher proportion in our cohorts of patients due to the inclusion of early cases, when greater surgical aggressiveness was still prevalent in our country, as well as in the early literature [29]. Therefore, the previously recommended surgical approach was total nephrectomy on the most affected side associated with partial nephrectomy in the contralateral kidney. This shifted over the years to the current approach of nephron-sparing surgery in patients with bilateral tumors or disease in a single kidney [30]. The results are quite satisfactory, showing that they do not compromise disease-free survival and allow for a better quality of life [31,32]. Moreover, some services have extended this approach to cases of unilateral tumors in non-syndromic children, finding favorable results in these cases as well, although careful consideration is required to balance the benefits of parenchymal preservation with the need for radiotherapy in unilateral NSS cases [33,34].

The preservation of healthy renal parenchyma is associated with a lower risk of clinical complications, such as renal failure or systemic arterial hypertension. In addition, the oncological outcome of this surgical technique is safe regarding similar recurrence rates (although it is difficult to determine whether it is a recurrence or a new tumor in patients with underlying predisposition) as compared to more aggressive procedures [35,36]. Therefore, nephron-sparing surgery is currently the gold-standard procedure after neoadjuvant chemotherapy worldwide [37], with several studies showing similar oncological results in well-selected patients [38,39,40,41,42,43]. In the present series of patients, this was the approach adopted in most patients with synchronous Wilms tumors. Several studies tried to assess the optimal surgical management using different techniques. The use of parenchymal cooling during nodulectomy, as evaluated by De Backer et al. (1995) [44] was shown to be ineffective and increased surgical time unnecessarily. Irtan et al. [45] highlight the importance of exploring new areas of improvement in specific subgroups of Wilms tumors, such as bilateral cases.

In the early phase of the casuistic, in a period when we used NWTS (National Wilms Tumor Study) and GBTW (Wilms Tumor Brazilian Group) protocols, some patients underwent bilateral biopsies after preoperative chemotherapy. However, we learned that if tumors do not respond to initial chemotherapy, even if there are still large masses bilaterally, the patient should be operated on. It is important to point out that even though preoperative images are helpful, they do not define tumor resectability or determine the need for total nephrectomy. The final decision on surgical treatment is made intraoperatively and the use of intraoperative ultrasound is helpful. Vascular extension can occur in such cases, and careful evaluation of images is required for surgical planning.

During a traditional partial nephrectomy, the blood supply to the part of the kidney being removed is clamped off to prevent bleeding. This period of ischemia can damage the remaining kidney tissue and potentially lead to complications. A surgical technique used to remove a tumor from the kidney while preserving as much healthy kidney tissue as possible with no vascular clamping of the renal hilum, thereby avoiding ischemic injury, even if transient, of the remaining healthy renal parenchyma, has been mainly described in minimally invasive surgery [46]. In this series, the local ischemia was obtained with digital compression of the renal parenchyma. This approach minimizes the risk of damage to the remaining healthy kidney tissue and reduces the likelihood of complications, such as acute kidney injury and bleeding. Most patients in this study underwent a surgical protocol involving a transperitoneal approach and lymph node sampling [47]. Although some groups of surgeons still use specific techniques during surgical management, such as autotransplant bench, use of hemostatic agents, and use of a ureteral catheter, these were not used in this series [48]. The approach used did not affect the outcome of these patients, and complication rates were consistent with those found in other techniques. Recently, a technical review was published describing each step of this critical procedure that uses marginal resection and direct compression to avoid bleeding, without vascular clamping, in line with the surgical approach described in the present series. This technique prevents injury to the normal adjacent parenchyma, and it is considered safe. In some cases, a type of sealant patch can be used to facilitate the hemostasis on the cut surface of the kidney [49]. Although the rate of microscopic positive margins is significantly higher in nephron-sparing approaches than in radical nephroureterectomy [50], the benefits of saving renal parenchyma despite a positive margin may outweigh the risks of the radiation therapy that would be required. In such cases, the overall survival and local recurrence rates are still excellent [16]. A survival curve analysis for margin status was made in the present series but was not consistent enough and could be biased by other staging factors.

Some patients may need a repeat NSS approach due to local relapse or metachronous tumor development, which is considered safe and feasible in the literature [51]. In our series, patients underwent from one to five surgical approaches. In two situations, the nephron-sparing approach is not indicated: tumors with vascular extension and tumors with diffuse anaplasia, when the surgical procedure must be completed with total nephrectomy, since the risk of recurrence becomes unacceptable [52,53].

Simultaneous intervention in both kidneys benefits the patient by reducing the chance of drug resistance, and morbidity related to a second surgical approach or a new pause in adjuvant treatment.

NSS is recommended in patients with unilateral WT and genetic predisposition due to the risk of metachronous contralateral tumor development and/or the long-term risk of renal failure. The Umbrella protocol allows for NSS in unilateral non-syndromic WT in cases in which the tumor is polar or peripheral, <300 mL in volume, without evidence of preoperative rupture, no intraluminal renal pelvic tumor, no adjacent organ invasion, no intravascular thrombus, no multifocality, and in which a negative margin with a renal remnant having greater than 66% of the original kidney volume is expected [54]. One case of the present series was excluded because the postoperative pathology revealed bilateral xanthogranulomatous pyelonephritis. Rates of misdiagnosis in Wilms tumor are around 4% in the literature [55]. A recent study in our institution found a diagnostic accuracy of 98.1%, which highlights the quality of the multidisciplinary team. Nonetheless, some pathologies can be misdiagnosed as WT, especially when they present with unspecified symptoms and dubious images [56].

The post-surgical clinical complications found were systemic arterial hypertension, acute renal failure, and chronic renal failure, the last resulting from bilateral nephrectomies, as reported in other studies, but with a lower prevalence rate [57]. There were no surgical complications in the patients in this sample, such as urinary fistula or severe intraoperative hemorrhage.

The overall mortality rate was similar to other studies, remaining in the range of 10–20% [16]. The same happened to the analysis of the disease-free survival rate, which remains at around 80% [58]. Currently, new technologies may help surgeons to decide on a better intraoperative strategy. Intraoperative ultrasound examination is useful for determining the boundaries of the tumor, the proximity to major vessels, and safe resection, for example [45].

As for future perspectives, fluorescence-guided surgery using indocyanine (ICG) can be useful to determine surgical margins intraoperatively for bilateral Wilms tumors [59,60,61,62,63].

Minimally invasive approaches, such as laparoscopic-assisted and robotic-assisted laparoscopy, have been reported mostly for unilateral tumors but have limitations for bilateral tumors [54,64,65,66,67].

The use of a three-dimensional printed model is a recent and innovative option that can aid surgeons in planning their approach, and better understanding the anatomic relation between the tumor and the normal kidney, in addition to the relationship to other structures of interest, like vascular structures in bilateral tumors [68,69,70,71]. A “virtual resection” can be experimented during a real-time manipulation of 3D reconstructions to plan NSS and to estimate the expected residual renal volume after resection [72].

Finally, the role of the metaverse was recently discussed. The fusion between the real and virtual worlds can create opportunities to improve surgical management in several respects, for example, helping parents to understand the surgical aspects or creating a path to take the spectator into the virtual operating theater, offering a new important tool for presurgical planning and counseling, with the additive values of overcoming distance and possible home usage [73].

In this series, there were no patients eligible for minimally invasive approaches, and the only technique used was intraoperative ultrasound to facilitate the dissection limits and confirm the position of intraparenchymal lesions.

## 5. Conclusions

The surgical management of Wilms tumor has a relevant role in contributing to a satisfactory outcome, especially in bilateral cases where, in addition to the curative objective, the quality of life after treatment must be considered. Given the rarity of bilateral cases, standardizing surgical techniques is challenging. Our evaluation of the surgical techniques used in our institution demonstrated comparable results in terms of complication rates and long-term survival. Digital compression of the parenchyma during the NSS approach allows adequate bleeding control, in addition to the low rate of urinary fistulas with only the use of open parenchyma suture, without the use of drains or double-J.

Furthermore, new technologies have the potential to enhance safety in surgical procedures and can contribute to better outcomes, greater rate of parenchymal preservation, and better quality of life.

## Figures and Tables

**Table 1:** Patient characteristics.

Patients Characteristics	Number (%)
Sex	
Boys	11 (33.3)
Girls	22 (66.7)
Total	33 (100)
Age at diagnosis	
<12 months	9 (27.3)
12–24 months	8 (24.2)
>24 months	16 (48.5)
Total	33 (100)
Initial symptoms	
Increased abdominal volume	16 (48.5)
Abdominal mass	11 (33.3)
Weight loss	2 (6.0)
Hematuria	4 (12.1)
Abdominal pain	3 (9.0)
Fever	3 (9.0)
Incidental	2 (6.0)
Constipation	2 (6.0)
Presentation	
Synchronous	30 (91.0)
Metachronous	3 (9.0)
Total	33 (100)
Metastases at diagnosis	
No	29 (87.9)
Yes	4 (12.1)
Total	33 (100)
Vascular extension	
Yes	1 (3.0)
No	32 (97.0)
Total	33 (100)
Anomalies	
Cryptorchidism	2 (6.0)
Beckwith–Wiedmann	2 (6.0)
Hemihypertrophy	3 (9.0)
Hypospadia	1 (3.0)
Horseshoe kidney	1 (3.0)
None	25 (75.7)

## Data Availability

The data presented in this study are available on request from the corresponding author.
